# Unique lymphocyte transcriptomic profiles in septic patients with chronic critical illness

**DOI:** 10.3389/fimmu.2024.1478471

**Published:** 2024-12-03

**Authors:** Evan L. Barrios, Leandro Balzano-Nogueira, Valerie E. Polcz, Christine Rodhouse, Jack R. Leary, Dijoia B. Darden, Jaimar C. Rincon, Marvin L. Dirain, Ricardo Ungaro, Dina C. Nacionales, Shawn D. Larson, Ashish Sharma, Gilburt Upchurch, Shannon M. Wallet, Todd M. Brusko, Tyler J. Loftus, Alicia M. Mohr, Robert Maile, Rhonda Bacher, Guoshuai Cai, Michael P. Kladde, Clayton E. Mathews, Lyle L. Moldawer, Maigan A. Brusko, Philip A. Efron

**Affiliations:** ^1^ Sepsis and Critical Illness Research Center, Department of Surgery, University of Florida College of Medicine, Gainesville, FL, United States; ^2^ Diabetes Institute, University of Florida, Gainesville, FL, United States; ^3^ Department of Biostatistics, University of Florida College of Medicine and Public Health and Health Sciences, Gainesville, FL, United States; ^4^ Department of Oral Biology, University of Florida College of Dentistry, Gainesville, FL, United States; ^5^ Department of Pathology, Immunology and Laboratory Medicine, University of Florida College of Medicine, Gainesville, FL, United States; ^6^ Department of Biochemistry and Molecular Biology, University of Florida College of Medicine, Gainesville, FL, United States

**Keywords:** lymphocytes, single-cell RNA sequencing, chronic critical illness, sepsis, human

## Abstract

**Introduction:**

Despite continued improvement in post-sepsis survival, long term morbidity and mortality remain high. Chronic critical illness (CCI), defined as persistent inflammation and organ injury requiring prolonged intensive care, is a harbinger of poor long-term outcomes in sepsis survivors. Current dogma states that sepsis survivors are immunosuppressed, particularly in CCI. Investigation of this immune suppression in heterogeneous immune populations across distinct clinical trajectories and outcomes, along with limited sampling access, is accessible via single-cell RNA sequencing (scRNA-seq).

**Methods:**

scRNA-seq analysis was performed on healthy subjects (n=12), acutely septic patients at day 4 ± 1 (n=4), and those defined as rapid recovery (n=4) or CCI (n=5) at day 14-21. Differential gene expression and pathway analyses were performed on peripheral blood lymphocytes at both a population and annotated cell subset level. Cellular function was assessed via enzyme-linked immunosorbent spot (ELISpot), cytokine production analysis, and T-cell proliferation assays on an additional cohort of septic patients (19 healthy, 68 acutely septic, 27 rapid recovery and 20 classified as CCI 14-21 days after sepsis onset).

**Results:**

Sepsis survivors that developed CCI exhibited proportional shifts within lymphoid cell populations, with expanded frequency of CD8^+^ and NK cells. Differential expression and pathway analyses revealed continued activation in T cells and NK cells, with generalized suppression of B-cell function. Both T and NK cell subsets displayed transcriptomic profiles of exhaustion and immunosuppression in CCI, particularly in CD8^+^ T effector memory (TEM) cells and NK cells. Functional validation of T-cell behavior in an independent cohort demonstrated T cells maintained proliferative responses *in vitro* yet exhibited a marked loss of cytokine production. IFN-γ production at the acute phase (day 4 ± 1) was significantly reduced in subjects later classified as CCI.

**Discussion:**

Sepsis patients exhibit unique T-, B-, and NK-cell transcriptional patterns that are both time- and clinical trajectory-dependent. These transcriptomic and pathway differences in sepsis patients that develop CCI are associated with exhaustion in CD8^+^ TEM cells and NK cells. Understanding the specific immune system patterns of different cell subsets after sepsis at a molecular level will be key to the development of personalized immunotherapy and drug-targeting intervention.

**Clinical trial registration:**

https://clinicaltrials.gov/, identifier NCT02276417.

## 1 Introduction

Increasingly, surgical sepsis patients are surviving their initial infectious insults. Although in-hospital mortality has declined due to earlier recognition of sepsis and improved implementation of best practices (1), chronic survival and restoration of functional and cognitive performance has been less successful. Long-term recovery appears to be determined, at least in part, by the duration and complexity of in-hospital clinical trajectories (2, 3). Importantly, sepsis survivors who do not rapidly recover but instead develop chronic critical illness [CCI, characterized by >14 days in the ICU with persistent organ dysfunction (4)] exhibit dismal outcomes and increased mortality (5). As approximately 1/3 of septic surgical patients experience CCI (1), recent research and clinical practices focus on early identification and potential interventions for patients destined to develop CCI.

Current dogma argues that sepsis survivors are immunosuppressed, characterized by an increased incidence of secondary infections and increased readmissions for sepsis after discharge (6, 7). In such patients, it is presumed that T-cell exhaustion results in loss of function followed by cell death (8, 9). B-cell and NK-cell dysfunction also occurs after sepsis, although this is less well understood (10). However, immune suppression in sepsis is complex and may not be uniformly exhibited across all cell types and cohorts of patients.

In an era of precision medicine, single-cell RNA sequencing (scRNA-seq) has emerged as a powerful tool to better understand the diversity of transcriptomic changes across different immune cell subsets in surgical sepsis patients (11). Only recently has this technology been applied to discriminating the immune response in sepsis patients with different clinical trajectories and outcomes (12, 13). Previous research has identified transcriptomic differences in myeloid cells between patients who develop CCI and those that rapidly recover after sepsis (12). Here, we have used scRNA-seq to examine the transcriptomes of individual lymphoid subsets after sepsis in survivors with different clinical trajectories and have compared a subset of those results to lymphocyte functional measures.

## 2 Materials and methods

### 2.1 Study design

The following study design is summarized from our previous publication (13). This prospective, observational cohort study was performed at a tertiary care, academic research hospital and is registered on clinicaltrials.gov (NCT02276417). The objective of the study was to identify unique transcriptomic signatures in various immune cell subsets in patients who developed CCI after surgical sepsis compared to those who rapidly recovered. Patients were classified as CCI if they had ICU length of stay ≥14 days with persistent organ dysfunction (as measured by the Sequential Organ Failure Assessment (SOFA) score) or ICU length of stay <14 days at this institution but were transferred to another hospital, long-term acute care facility (LTAC), or hospice with persistent organ dysfunction (14, 15). Patients who otherwise recovered were classified as rapid recovery. Additionally, cells were analyzed at two different post-sepsis time points (i.e., day 4 ± 1 and days 14-21).

Sepsis was identified as previously described using Sepsis-3 definitions (9). In short, an existing electronic medical record-based system was utilized (the Modified Early Warning Signs-Sepsis Recognition System, or MEWS-SRS). Once identified via this system, patients with sepsis were treated with protocolized care based on the Surviving Sepsis guidelines (9). Inclusion and exclusion criteria consisted of the following:

Inclusion criteria:
  Admission to the intensive care unit (ICU)
  Age >17 years
  Diagnosis of sepsis or septic shock according to the 2016SCM/ESICM International Sepsis DefinitionsConference [Sepsis-3] (9)
  Initial septic episode while hospitalized
  Management of patient via the sepsis clinicalmanagement protocol (16)
Exclusion criteria:
  Refractory shock
  Inability to achieve source control
  Pre-sepsis expected lifespan <3 months
  Expected withdrawal of care
  Severe congestive heart failure (NYHA Class IV)
  Child-Pugh Class C liver disease or undergoingevaluation for liver transplant
  HIV infection with CD4^+^ count <200 cells/mm^3^
  Prior organ transplant, use of chronic steroids, orimmunosuppressive agents
  Pregnancy
  Institutionalized or other vulnerable patient populations
  Chemotherapy or radiotherapy treatment within 30days of sepsis onset
  Severe traumatic brain injury (defined by radiologicevidence and GCS <8)
  Spinal cord injury with permanent deficits
  Unable to obtain informed consent

### 2.2 Human sample collection

For scRNA-seq, power calculations were previously determined in the pilot study (11). Whole blood samples were collected from a total of 25 de-identified subjects in the following cohorts: healthy subjects (n=12), acutely septic patients (n=4, collected 4 ± 1 days after sepsis onset), and samples from patients 14-21 days after sepsis (divided into patients with CCI [n=5] and those who rapidly recovered [n=4]). All patients had samples collected at the day 14 time point, which was used for scRNA-seq (see below). All acutely septic patients met criteria for septic shock (9). All septic cohorts were similar in underlying comorbidities and age (although healthy subjects trended towards being younger than the sepsis patients) (**Table 1**). Following sample collection, peripheral blood mononuclear cells (PBMCs) were collected using Ficoll-Paque™ PLUS (Millipore Sigma, St. Louis, MO) and density gradient centrifugation.

**Table 1:** Patient characteristics between cohorts.

	Healthy Subjects (n=12)	Sepsis Day 4 ± 1 (n=4)	RAP Days 14-21 (n=4)	CCI Days 14-21 (n=5)	p-value
Male, # (%)	7 (58)	1 (25)	1 (25)	3 (60)	0.48
Age in years, (μ ± SD)	46 ± 10	67 ± 22	61 ± 16	58 ± 18	0.08
BMI (μ ± SD)		39 ± 19	37 ± 20	21 ± 3	0.19
Septic shock, # (%)		4 (100)	1 (25)	4 (80)	
Pressor requirement (%)		4 (100)	3 (75)	4 (80)	
Steroid use (%)			1 (2)	0 (0)	
CCI (median)		5.5	2	2	
Comorbidities (#)	Cancer (1), COPD (1), DM (1), HTN (3)	COPD (1), DM (2), HTN (4)	COPD (1), DM (1), HTN (4)	DM (1), HTN (2)	
Admission Diagnosis (#)		NSTI (1), Choledocholithiasis (1), SBO (1), Planned operation (1)	NSTI (2), SBO (2)	Planned operation (1), Complication (1), Intra-abdominal abscess (1) Pancreatitis (1), MCC (1)	
Site of infection (#)		NSTI (2), cholecystitis (1), intra-abdominal (1)	UTI (1), pneumonia (1), NSTI (2)	NSTI (1), anastomotic leak (1), prosthetic infection (1), pneumonia (1), intra-abdominal (1)	
Pathogens isolated (#)			Klebsiella (1), candida (1), enterobacter (1)	Candida (1), staph aureus (1), strep viridins (1)	
Organ dysfunction (#)		Renal dysfunction (1), altered mentation (1)	Respiratory failure (2), altered mentation (1)	Altered mentation (2), Acute renal failure (2), Respiratory failure (2), ARDS (1)	

Cohorts consist of healthy control patients, acutely (day 4 ± 1) septic patients, and late (days 14-21) sepsis patients who experienced rapid recovery (RAP) and chronic critical illness (CCI). There were no significant differences in sex, age, or BMI between the groups. BMI, body mass index; CCI, Charlson comorbidity index; COPD, chronic obstructive pulmonary disease; DM, diabetes mellitus; HTN, hypertension; NSTI, necrotizing soft tissue infection; SBO, small bowel obstruction; MCC, Motorcycle crash. Adapted from Barrios et al. (12).

For T-cell proliferation analysis, cytokine production, and ELISpot analysis, whole blood samples were collected in a similar fashion to patients included for scRNA-seq analysis in the following cohorts: healthy subjects (n=19), acutely septic patients at day 4 ± 1 (n=68), and samples from patients 14-21 days after sepsis onset (CCI n=20, rapid recovery n=27). Some patients contributed samples while both acutely septic and 14-21 days after sepsis onset. The acutely septic patients were followed by chart review in order to determine if they rapidly recovered or if they entered CCI. Healthy subjects were significantly younger than the septic patients (healthy mean age= 46, sepsis mean age=61, p=0.0001). Not all samples underwent all forms of functional analysis testing.

Patients of both sepsis cohorts were admitted to the same institution and enrolled in studies with overlapping inclusion criteria. The power analysis for T-cell cytokine production for IL-4 and IL10 showed low probability (54.5% and 30.7%, respectively) values; however, despite the small sample size (minimum n=7), high power probability was achieved for IFN-γ (87.6%) to detect a true difference between these sepsis cohorts. Our approach is adequate to identify significant differences between these groups, based on a two-sample two-tailed t-test with α = 0.05.

### 2.3 scRNA-seq

#### 2.3.1 scRNA-seq reads pre-processing

PBMCs were encapsulated for scRNA-seq using the 10x Genomics platform. Specifically, gene expression and feature-barcoding data were generated using 10x Genomics v1.1 5’ chemistry and were sequenced on an Illumina HiSeq with a target of 5,000 cells per sample (17). Cell Ranger software suite was used to process the base calls into FASTQ files, which were then checked for quality control aberrations using FastQC v0.11.7 (18). A spliced + intronic, or *splici*, reference transcriptome was generated from the hg38 reference genome (19). Reads were pseudo-aligned to the reference transcriptome with alevin-fry v0.8.1; USA mode was used for the gene expression reads in order to provide separate quantifications of spliced, unspliced, and ambiguous mRNA abundance (20–22). The counts of 11 cell surface proteins of interest were also quantified using alevin-fry. Splicing-aware gene expression quantification was mapped to Ensembl transcript IDs, with final count matrices aggregated using Ensembl gene IDs.

#### 2.3.2 scRNA-seq data processing

Downstream data processing and analysis were performed primarily in R v4.3.1, with some additional processes being written in Python v3.10 as required (23, 24). After loading the unfiltered spliced, unspliced, and ambiguous mRNA counts into R using the fishpond package v2.4.1, we defined total mRNA counts as the elementwise sum of all three counts matrices and added the ambiguous counts to the spliced counts matrix (25). Unless otherwise specified, total mRNA counts were used as input throughout the analysis. Doublets were then identified and filtered out using the DropletUtils package v1.18.1 (26, 27). Cells with an estimated false discovery rate (FDR) of <0.01 were kept for each sample. Next, the percentage of spliced reads coming from mitochondrial genes was computed for each cell, and cells with less than 5% mitochondrial DNA were kept. The final merged dataset was composed of 36,601 genes and 66,225 cells.

#### 2.3.3 Dimensionality reduction, normalization and data integration

The read counts for each gene were log-normalized based on Ward’s distance (28). Next, the 25 samples were integrated by the Harmony package v1.2.0 (29). Then, the first 50 principal components were used as input to generate UMAPs (30). Lastly, clusters were generated via Louvain modularity optimization using a resolution of 0.1 and a number of evaluated nearest neighbors of 100 (**Supplementary Figure S1A**) (31).

#### 2.3.4 scRNA-seq cell annotation

After clustering, multiple methods were executed to perform the cell annotation in SingleR v2.4.1 and Azimuth 0.4.6.9004. Both packages perform cell identification by matching individual cells to a reference dataset of known cell types. Then, a set of informative genes that distinguish different cell types was generated. SingleR was employed to build a predictive model using selected genes from the reference dataset via a single-sample gene set enrichment analysis (ssGSEA) approach. Each cell in the target dataset was then annotated with a predicted cell type label from the reference dataset (32). A total of 5 different reference datasets were used to annotate the cells: Blueprint ENCODE data, 259 RNA-seq samples of pure stroma and immune cells (33, 34); Database Immune Cell Expression Data (DICE), 1561 bulk RNA-seq samples of sorted cell populations (35); Human Primary Cell Atlas Data, a community that has annotated millions of human cells from many different tissues (36); Monaco Immune Data, 114 bulk RNA-seq samples of sorted immune cell populations that can be found in GSE10701110 (37); Novershtern Hematopoietic Data, comprising 211 bulk human microarray samples of sorted hematopoietic cell populations that can be found in GSE2475911 (38). Azimuth was also used to annotate, utilizing a publicly available healthy human PBMC reference with 161,764 cells (39). Cells were annotated by consensus of 5 out of 6 (>0.80) annotation procedures. This allowed for more homogenous cell annotation at a higher resolution of classification (**Table 2**). Only lymphoid cells were retained for subsequent analyses, resulting in 18,851 cells (1,451 B cells, 11,550 CD4^+^ T cells, 2,371 CD8^+^ T cells, 3,161 NK cells and 318 other T cells).

**Table 2:** Relative cell frequencies by cohort.

		Healthy Subjects (n=12)	Sepsis Day 4 ± 1 (n=4)	RAP Days 14-21 (n=4)	CCI Days 14-21 (n=5)	Total
B cells	B naive	251 (2.04)	8 (0.77)	93 (3.82)	84 (2.76)	436
	B intermediate	178 (1.44)	7 (0.67)	39 (1.60)	28 (0.92)	252
	B memory	140 (1.14)	3 (0.29)	55 (2.26)	17 (0.56)	215
	Plasmablast	73 (0.59)	97 (9.29)	198 (8.13)	179 (5.88)	547
CD4+ T	CD4^+^ T naïve	1936 (15.71)	56 (5.36)	21 (0.86)	305 (10.02)	2318
	CD4^+^ T proliferating	20 (0.16)	7 (0.67)	13 (0.53)	3 (0.10)	43
	CD4^+^ TEM	157 (1.27)	11 (1.05)	22 (0.90)	33 (1.08)	223
	CD4^+^ TCM	5683 (46.10)	676 (64.75)	1290 (52.96)	1042 (34.23)	8691
	CD4^+^ CTL	11 (0.09)	0 (0.00)	3 (0.12)	7 (0.23)	21
	Treg	149 (1.21)	16 (1.53)	24 (0.99)	36 (1.18)	225
CD8+ T	CD8^+^ T naïve	401 (3.25)	6 (0.57)	20 (0.82)	118 (3.88)	545
	CD8^+^ T proliferating	10 (0.08)	0 (0.00)	11 (0.45)	8 (0.26)	29
	CD8^+^ TEM	926 (7.51)	80 (7.66)	146 (5.99)	453 (14.88)	1605
	CD8^+^ TCM	153 (1.24)	4 (0.38)	14 (0.57)	24 (0.79)	195
Other T cells	dnT	60 (0.49)	5 (0.48)	21 (0.86)	10 (0.33)	96
	gdT	117 (0.95)	10 (0.96)	12 (0.49)	7 (0.23)	146
	MAIT	64 (0.52)	1 (0.10)	12 (0.49)	21 (0.69)	98
NK	NK	1815 (14.72)	47 (4.50)	381 (15.64)	595 (19.55)	2838
	NK Proliferating	60 (0.49)	5 (0.48)	18 (0.74)	52 (1.71)	135
	NK CD56^bright^	123 (1.00)	5 (0.48)	22 (0.72)	43 (1.77)	193
Total		12326 (100.00)	1044 (100.00)	3044 (100.00)	2436 (100.00)	18851

Reported as absolute cell number (relative proportion). RAP, rapid recovery; CCI, chronic critical illness; TEM, T effector memory cell; TCM, T central memory cell; CTL, cytotoxic T lymphocytes; Treg, regulatory T cells. dnT, double negative T cells; gdT, gamma delta T cells; MAIT, mucosal-associated invariant T cells; NK, natural killer cells.

#### 2.3.5 Differential expression analysis, functional enrichment, and pathway analysis

Differentially expressed (DE) genes were identified after stratification by cell type using a mixed-effects model implemented in Julia (40). In all analyses, the rapid recovery subjects were considered as the reference group and CCI subjects considered the comparison group. Mixed-effects models are particularly useful when analyzing data that exhibit hierarchical or grouped structure, as in our case where cells are nested within individual subjects. These models account for both fixed effects, which capture population-level effects (such as group differences), and random effects, which account for variability at the individual subject level. By incorporating random effects, mixed-effects models allow for more accurate inference, as they adjust for correlations between cells from the same individual and reduce the risk of inflated significance that can arise from treating all cells as independent observations, which are especially adequate when dealing with low sample size. The mixed-effects model was employed to assess the differential expression of genes across various conditions, while controlling for individual variability. This approach provides a robust framework for identifying DE genes in datasets where observations are not independent, such as single-cell data from different patients. The code used for the analysis can be accessed at https://github.com/leobalzano/MixedModelsJuliaCall.R.

The *p*-values were adjusted for multiple testing using the FDR method. Due to the relatively low sample size and the desire to perform pathway enrichment analyses, each cell type comparison required a different adjusted p-value threshold for the genes to be considered as differentially expressed. The threshold used was as follows: *p*_adj_<0.5 for B cells, *p*_adj_<0.1 for CD8^+^ T cells, and *p*_adj_<0.01 for NK and CD4^+^ T cells. Functional enrichment and pathway analyses were performed using the DE genes utilizing five databases (DAVID, GO terms, KEGG pathways, Reactome, and CORUM). The enriched pathways were selected by Fisher’s exact test with multiple-testing correction (*p*_adj_<0.05) (41).

### 2.4 Functional analysis of lymphoid cells

#### 2.4.1 Statistical approach for exhaustion test

For assessing cellular exhaustion, a linear mixed-effects model was applied to evaluate differences in exhaustion-related gene expression between patient cohorts. Specifically, we calculated module scores for a set of exhaustion-associated genes in individual cells from CD8+ TEM and NK cells using the AddModuleScore function in the Seurat package (42). The module scores were then used as the dependent variable in a mixed-effects model, with cell type and patient condition as fixed effects, and donor identity as a random effect to account for inter-donor variability. This approach allows for cell-level modeling, maintaining the complexity of the single-cell data while controlling for donor-specific effects.

#### 2.4.2 Human T-cell isolation and proliferation assay

These methods were previously reported in Barrios et al. (12). Briefly, following PBMC isolation, T cells were further isolated by immunomagnetic negative selection using EasySep™ Human T-Cell isolation kit (STEMCELL Technologies, Vancouver). CD3^+^ lymphocytes were labeled with Cell Trace violet (Thermo Fisher, Waltham, MA) in order to assess T-cell proliferation by dye dilution. T lymphocytes (1 x 10^5^ CD3^+^) were seeded into a 96-well plate and either stimulated with soluble anti-CD3/CD28 antibodies (STEMCELL Technologies, Vancouver) or left unstimulated. After 4 days, cells were harvested. The division index was determined as the total number of divisions divided by the number of cells at the start of the culture calculated via FlowJo™ Software (Becton, Dickinson and Company, Oregon).

#### 2.4.3 Cytokine analysis

Following a 4-day incubation at 37°C and 5% CO_2_, supernatants were obtained from the plates for cytokine analysis. Human high sensitivity T cell magnetic bead 8-plex panels (IFN-γ, IL-4, IL-10, IL-12 (p70), IL-17a, IL-2, TGFβ, and IL-23) were used (EMD Millipore, Billerica, MA) along with xPONENT software for cytokine analysis (EMD Millipore, Billerica, MA). T-cell cytokine production and ELISpot cohort medians were compared via Kruskal-Wallis non-parametric tests, with *p*<0.05 considered significant.

#### 2.4.4 Enzyme-linked immunosorbent spot assay

Diluted whole blood (50 μL) was used in order to measure the production of IFN-γ *ex-vivo* in stimulated and unstimulated cells following overnight culture using single-color ELISpot kits (ImmunoSpot, Cellular Technology Limited, Cleveland, OH). 96-well plates that were pre-coated with capture antibody were used for single color enzymatic assays. Plates were prepared with stimulant diluted in CTL-Test™ Medium (CTLT-005; Cellular Technology Limited, Cleveland, OH) and were incubated at 37°C and 5% CO_2_ for 30 minutes prior to cell plating. IFN-γ production was induced via a combination of 500 ng/mL of anti-human CD3 (clone HIT3a; BioLegend, San Diego, CA) and 5 μg/mL of anti-human CD28 (clone CD28.2; BioLegend, San Diego, CA).

Cells were incubated overnight following plating. A biotinylated secondary detection antibody, streptavidin-bond alkaline phosphatase and developer solution were applied to the samples. Finally, plates were air-dried in a laminar flow hood prior to image capture. Samples were analyzed for spot counts and spot size of IFN-γ using ImmunoSpot S6 Entry Analyzer with ImmunoSpot 7.0.30.4 professional software (CTL Analyzers, Cleveland, OH).

## 3 Results

### 3.1 Gene expression and pathway analysis suggest heterogeneous lymphoid dysfunction

**Figure 1:**
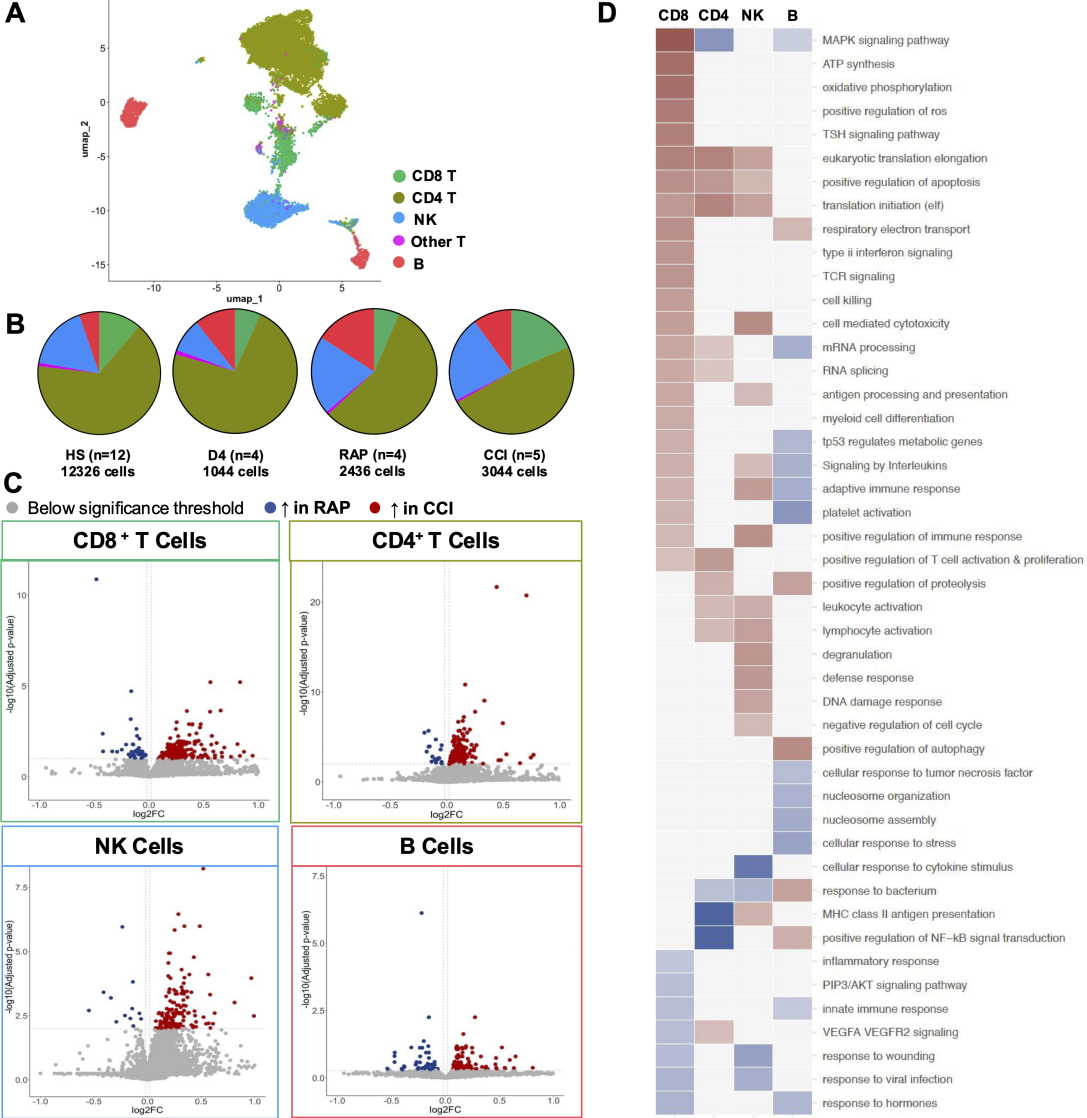
Single-cell RNA sequencing of the lymphoid compartment. **(A)** Uniform Manifold Approximation and Projection (UMAP) of 18,850 total lymphoid cells in all patient cohorts. **(B)** Cell type proportions and total cell numbers for each cohort. **(C)** Differentially expressed genes from the comparison between CCI and RAP were identified in CD8 T cells, CD4 T cells, NK cells, and B cells and subsequently used for pathway analysis. **(D)** Pathways passing significance threshold are displayed, with those upregulated in red, downregulated in blue. Color shading indicates p value. NK, natural killer cell.

In a combined cohort containing predominantly healthy subjects (**Table 1**) to enable robust cell annotation, lymphoid cells were isolated and assessed by scRNA-seq. Cells passing QC filters were integrated and broadly annotated (**Figure 1A**; **Supplementary Figure S1**) as CD4^+^ T cells, CD8^+^T cells, NK cells, innate-like “other” T cells, and B cells. We next determined the average proportional frequencies of cells within the dataset (**Figure 1B**) from the different septic cohorts. Given the differential abundance of the major lymphoid populations, and the paucity of innate-like T cells, we then focused our analyses on CD8 and CD4 T cells, NK cells, and B cells to observe their transcriptomic profiles.

Building on previous studies comparing septic patients to healthy subjects (11), we sought to explore differences in gene expression profiles between patients that developed CCI compared to those adjudicated as rapid recovery. DE analyses in annotated CD8^+^ T, CD4^+^ T, NK, and B cells were thus performed (**Figure 1C**; **Supplementary Material S1**). CD8^+^ T cells had 239 significantly differentially expressed genes (upper left panel, 197 up- and 42 downregulated). CD4^+^ T cells had 279 significantly differentially expressed genes (upper right panel, 262 up- and 17 downregulated). There were 168 significantly differentially expressed NK cell genes (bottom left panel, 156 up- and 12 downregulated). B cells had 189 significantly differentially expressed genes (bottom right panel, 131 up- and 58 downregulated).

Pathway analysis was then performed on the DE genes for each cell type (**Figure 1D**). CD8^+^ T cells displayed signs of heightened responsiveness in patients with CCI, as evidenced by enrichment in pathways associated with immune response, oxidative stress, and energy synthesis. CD4^+^ T cells exhibited a mixed immune response with enriched activation and proliferation pathways, but reductions in NF-κB and MAPK signaling pathways. Upregulated pathways in NK cells included effector functions such as cytotoxicity, degranulation, and activation. These cells also demonstrated reductions in response pathways to cytokine stimulus, bacteria, and viruses, indicating dysfunction within these cells. B cells generally displayed dampened immune response potential, with downregulated pathways in both innate and adaptive immune response, response to TNF, hormones and interleukins, and stress responsiveness. These results highlighted the heterogeneity of cell behaviors within the lymphoid compartment in response to sepsis, and merited deeper exploration of defined cell subsets within these major populations.

### 3.2 Alteration in lymphoid subset frequencies

**Figure 2:**
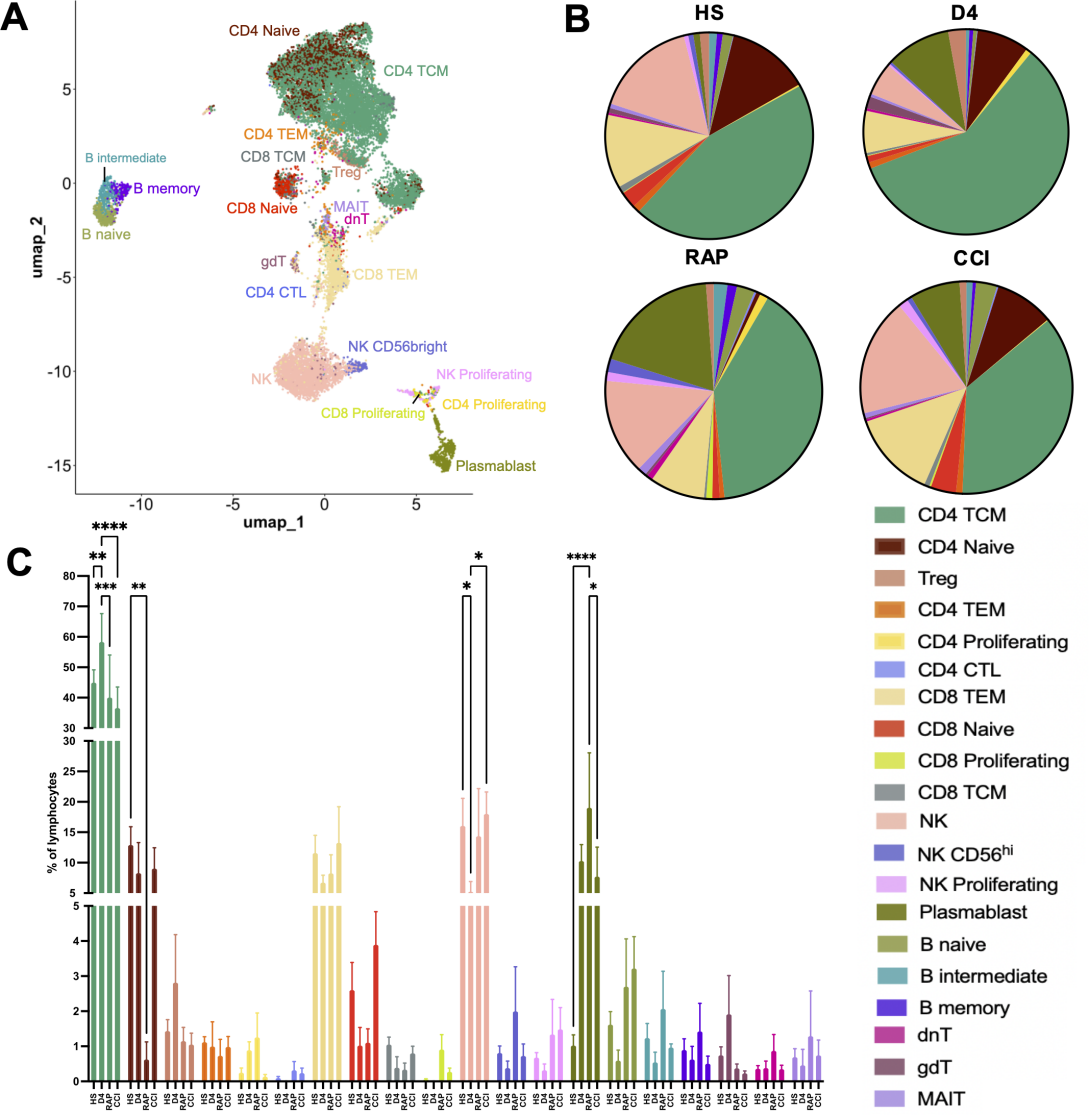
Lymphoid cell subset frequencies are altered during sepsis. **(A)** UMAP of cell clustering at higher annotation resolution to identify lymphoid cell subtypes. **(B)** Mean frequencies of cell populations identified in A displayed by group. **(C)** Between-group comparisons of cell subset frequencies within major lymphoid subsets across disease status groups. Cell frequencies reported as number of each cellular subtype divided by the total number of lymphoid cells per subject. HS: healthy subjects (n=12), D4: acutely septic subjects sampled at day 4 ± 1 (n=3), RAP, rapid recovery (n=3); CCI, chronic critical illness (n=5). NK, natural killer cells; TCM, central memory T cells; TEM, effector memory T cells; Treg, regulatory T cells; MAIT, mucosal-associated invariant T cells; dnT, double negative T cells; gdT, gamma delta T cells; CTL, cytotoxic T cells. *p<0.05, **p<0.01, ***p<0.001, ****p<0.0001; Two-way ANOVA with Tukey’s multiple comparison testing.

Consensus cell type annotations and determination of cell subset frequencies revealed an impact of disease status on cell abundance (**Figure 2**; **Supplementary Figure S2**). Twenty cellular subtypes were identified (**Figure 2A**), which were subsequently compared across the cross-sectional patient cohorts (**Figures 2B, C**). Multiple cell types exhibited altered frequency, with early (day 4 ± 1) septic subjects displaying signs of generalized lymphopenia, as previously described (43, 44). However, CD4^+^ central memory (CD4 TCM, **Figure 2C**, teal) cells were increased in early septic subjects, with a decrease in late (days 14-21) septic patients who rapidly recovered or developed CCI. Naïve CD4^+^ T cell frequencies (**Figure 2C**, brown) were increased in healthy subjects compared to rapid recovery patients (*p* < 0.01). We also observed lower NK cell frequencies (**Figure 2C**, salmon) in acutely (day 4 ± 1) septic patients compared both to healthy subjects and to patients with CCI, consistent with prior published literature (45). Overall, B cell frequencies displayed little variation between septic patients with CCI or those who rapidly recovered. Plasmablasts were increased in all subjects post-sepsis compared to healthy subjects, but only reached significance in rapid recovery subjects (**Figure 2C**, olive). CCI subjects had generally elevated plasmablast frequencies, yet they were distinctly lower in patients with CCI compared to rapid recovery (*p* < 0.05). Next, in order to define the context of the phenotypic state of the annotated cell subsets in sepsis subjects with CCI or rapid recovery, we performed DE analysis (**Supplementary Figure S3**). The genes identified largely reflected continued/chronic immune activation in those cells with altered frequency.

### 3.3 scRNA-seq of lymphoid immune cells reveals immune exhaustion in patients with CCI

**Figure 3:**
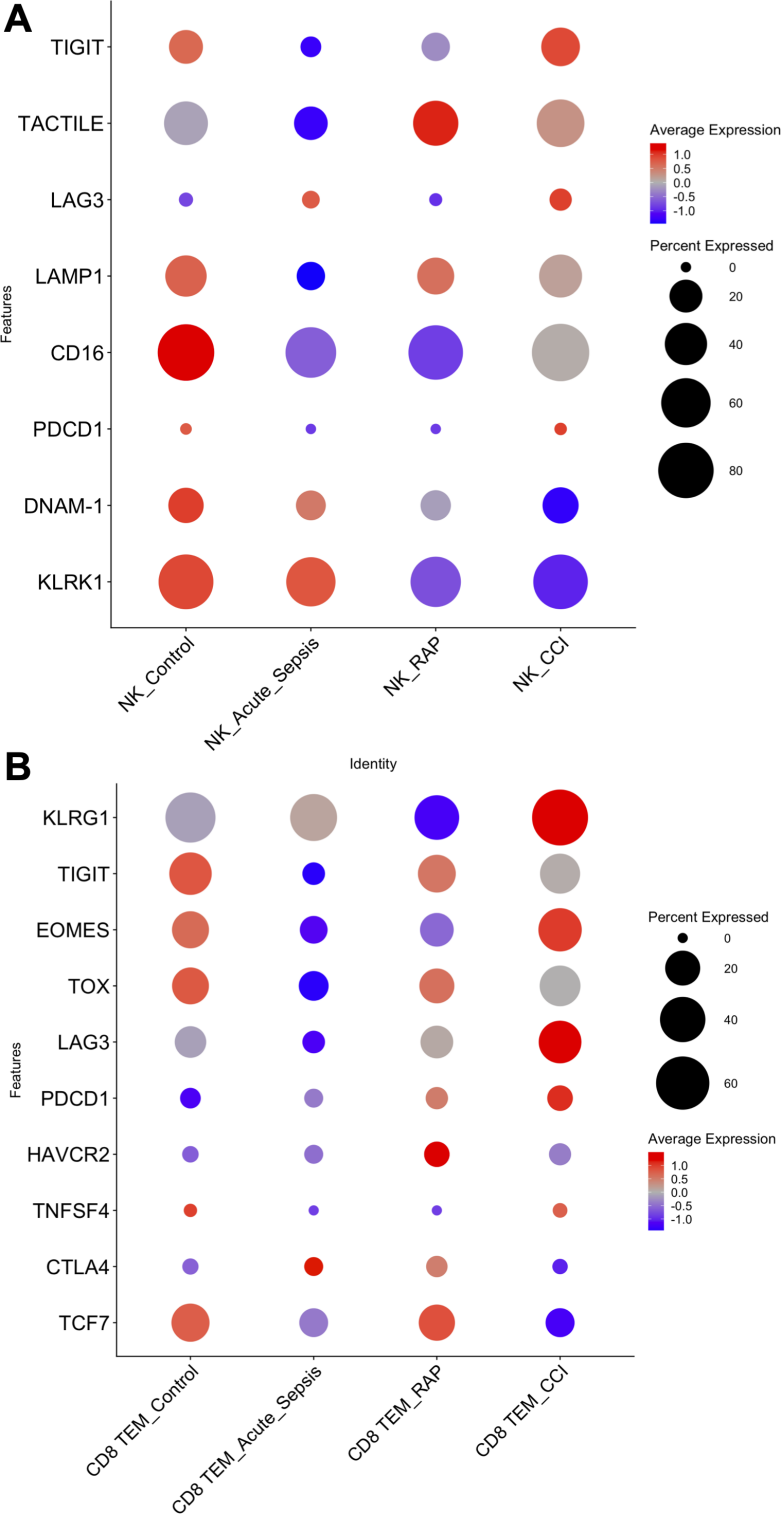
Assessment of cell exhaustion by gene expression in natural killer cells (NK) and CD8^+^ T effector memory (TEM) cells. Dot plots of genes associated with **(A)** NK and **(B)** CD8^+^ TEM cell exhaustion by patient cohorts. Genes reported in Roe et al. (45). Statistical analysis was performed using a linear mixed-effects model, with donor identity as a random effect and patient condition as a fixed effect. Only the comparison between CD8+ TEM cells from Control vs. CCI showed a statistically significant difference (p < 0.05). No significant differences were detected in NK cells, although a trend toward exhaustion was observed in CCI patients (p= 0.22). Control, healthy subjects; RAP, rapid recovery; CCI, chronic critical illness.

Having identified consistent *KLRG1* signatures in lymphoid cell types revealing upregulation of pathways related to stress and apoptosis, and the extensive overlap of these pathways with exhaustion, we subsequently analyzed exhaustion-associated genes in lymphoid cells. Specifically, we looked to identify if specific genes were upregulated in mature cell subtypes in patients with CCI compared to other cohorts, using a published reference as a guide (46). In NK cells, we observed downregulation of the effector molecules *DNAM-1* (DNAX accessory molecule-1) and *KLRK1* (killer cell lectin like receptor K1), along with upregulation of the negative regulators *TIGIT* (T-cell immunoreceptor with immunoglobulin and ITIM domain), *TOX* (thymocyte selection-associated HMB bOX), *TACTILE* [T-cell activation, increased late expression; identified as a negative regulator of NK cell function in mice, although the current function in humans is unknown (47)], *LAG3* (lymphocyte-activation gene 3), and *LAMP1* (lysosomal-associated membrane protein 1) (**Figure 3A**). In contrast, *CD16* (Fc gamma receptor III) expression was not significantly altered in NK cells (**Figure 3A**). All of the former is consistent with NK cells in CCI patients demonstrating exhaustion (46), however, despite a trend towards exhaustion in CCI patients (p = 0.22), there was not a statistically significant difference between the cohorts. Because CD8^+^ TEM cells were present in sufficient numbers, we were able to determine that they also displayed a gene expression profile [*KLRG1, TCF7, TNFSF4, CTLA4, TIGIT, EOMES, TOX, LAG3, PDCD1*, and *HAVCR2* (48, 49)] associated with cellular exhaustion, with a statistically significant difference between CCI patients and healthy controls (p < 0.05) (**Figure 3B**). Gene expression in CD4^+^ TEM cells, CD4^+^ TCM cells, B memory cells, and CD8^+^ TCMs from CCI patients was not consistent with cellular exhaustion (**Supplementary Figure S4**). These observations led us to investigate T-cell stimulus response and effector function *in vitro.*

### 3.4 T cell cytokine analysis revealed decreased production in patients with CCI

**Figure 4:**
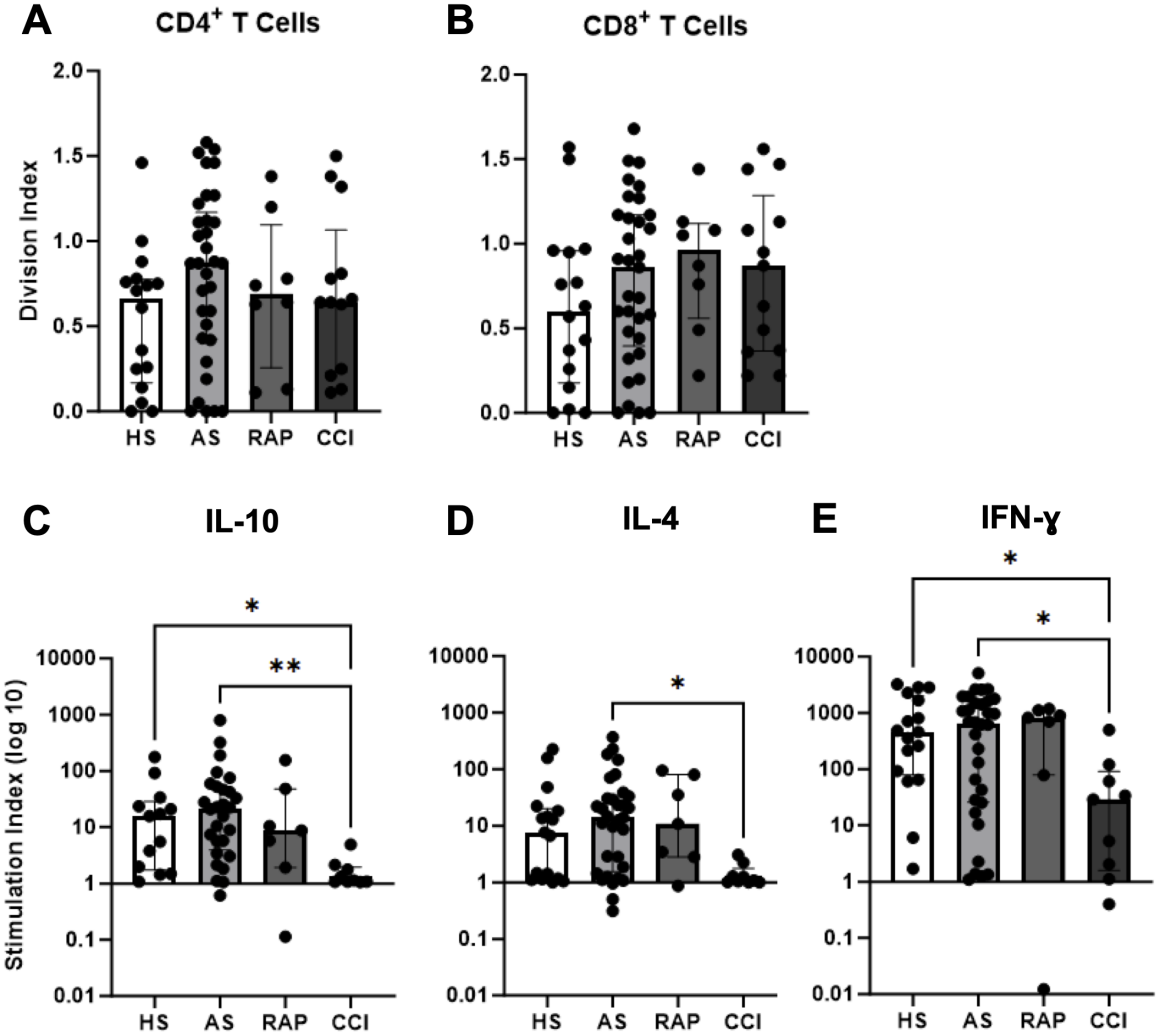
Functional proliferation and dysfunctional cytokine production in T cells. T cell proliferation was assessed by flow cytometry and division indices were calculated for gated CD4^+^ T cells **(A)** and CD8^+^ T cells **(B)** in healthy subjects (HS, n=16), acute sepsis (AS, n=33), rapid recovery (RAP, n=8), and chronic critical illness (CCI, n=13). **(C–E)** Multiplex cytokine analysis of culture supernatants for indicated cytokines compared across groups *p<0.05, **p< 0.01. Kruskal-Wallis test used for statistical comparison.

T cell proliferation and cytokine production in response to T-cell receptor stimulation were assessed *in vitro*. Although the scRNA-seq showed some significant downregulation of activation pathways in CD4^+^ T cells, interestingly, we found no significant difference in either CD4^+^ or CD8^+^ T cell proliferation in all septic subjects when compared to healthy subjects by division index (**Figures 4A, B**) (50). However, when considering cytokine production by stimulated T cells, significantly reduced levels of IL-10, IL-4, and IFN-γ were seen in patients with CCI compared to acutely septic patients at Day 4 ± 1, as well as significantly reduced IL-10 and IFN-γ in patients with CCI compared to healthy subjects (**Figures 4C–E**). This maintained proliferative response with a loss of cytokine production capacity is consistent with T cell progression to exhaustion.

### 3.5 ELISpot in peripheral blood reveals reduced IFN-γ in CCI

**Figure 5:**
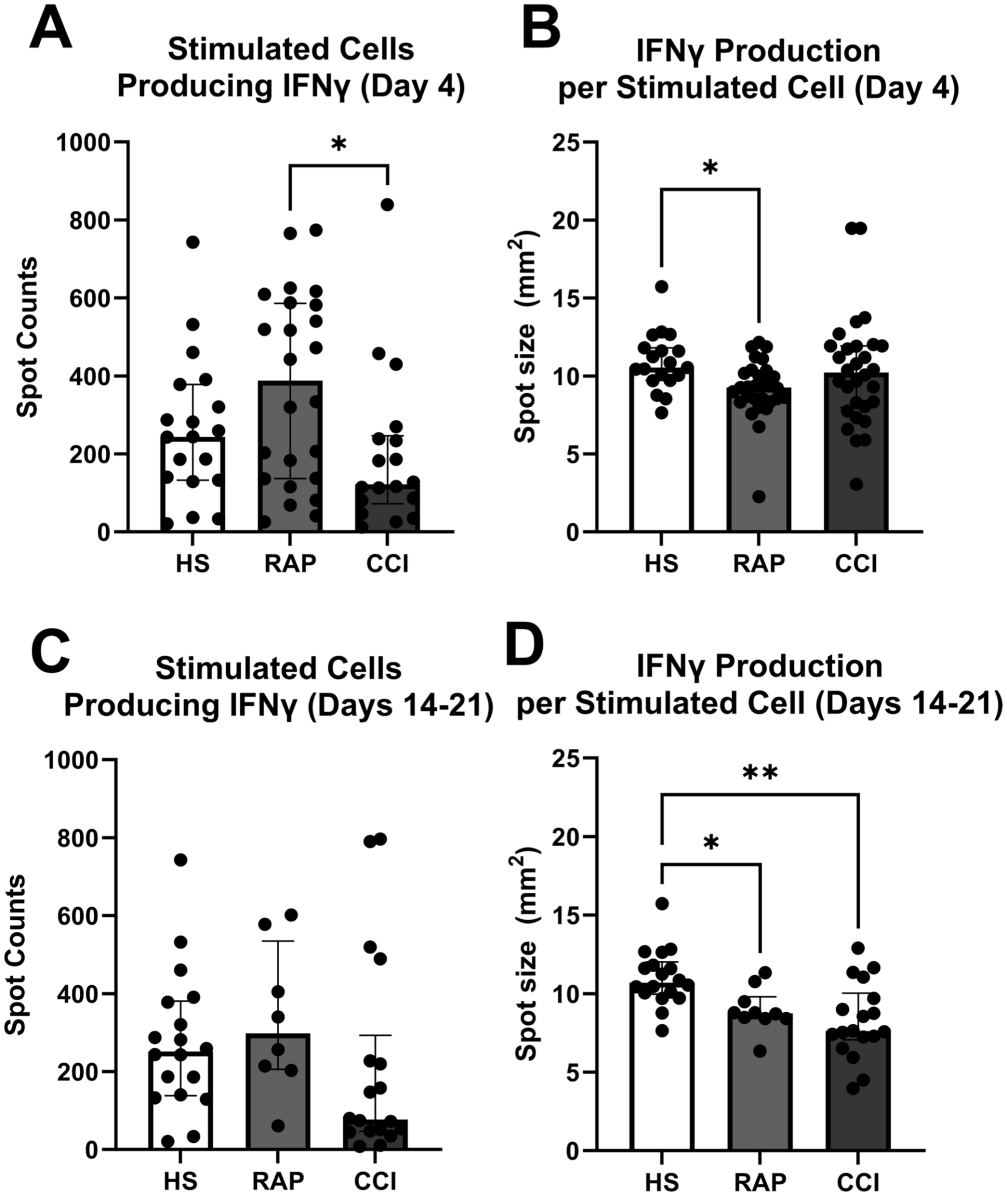
Enzyme-linked immunosorbent spot (ELISpot) quantification of IFN-γ production. **(A)** Number of stimulated cells producing IFN-γ sampled during acute phase (Day 4) [n=24 adjudicated as rapidly recovered (RAP), n=18 patients adjudicated as chronic critical illness (CCI)]. Healthy subjects serve as control (n=19). **(B)** IFN-γ production per cell in samples collected at day 4. **(C, D)** samples collected at 14-21 days after onset of sepsis (n=7 rapid recovery, n=13 chronic critical illness). Healthy subjects again serve as control (n=19). *p<0.05, **p<0.01, HS, healthy subjects; RAP, rapid recovery; CCI, chronic critical illness. Kruskal-Wallis test used for statistical comparison.

In peripheral whole blood collected from the same cohort of patients who underwent T-cell proliferation and cytokine analysis, there were fewer cells producing IFN-γ at post-sepsis day 4 ± 1 (**Figures 5A, B**) than in patients that entered CCI at days 14-21 as compared to patients that rapidly recovered (*p*=0.03) (**Figure 5A**). Patients sampled at day 4 ± 1 that subsequently entered rapid recovery, produced less IFN-γ per cell after stimulation *versus* healthy subjects (*p*=0.02) (**Figure 5B**). Interestingly, when the same stimulus response assay was performed at 2-3 weeks post-sepsis onset (**Figures 5C, D**), patients that progressed to CCI did not have fewer cells producing IFN-γ *versus* patients who rapidly recovered (*p*=0.1) (**Figure 5C**). However, cells from sepsis patients that developed CCI and experienced rapid recovery (days 14-21) produced less IFN-γ per cell after *ex vivo* stimulation *versus* healthy subjects (**Figure 5D**). These results indicate early and persistent dysfunctional stimulus response in septic subjects.

## 4 Discussion

Previous transcriptomic analyses of lymphoid cells after sepsis demonstrated unique patterns of gene expression in sepsis survivors (5). However, despite significant efforts to elucidate the mechanisms underlying sepsis in humans, functional differences in the lymphoid compartment are not well understood (51–53). Research has focused predominantly on CD4^+^ T cells, resulting in an enhanced understanding of their phenotype in septic subjects, characterized by an initial lymphopenia followed by alterations in subtype frequency, decreased cellular diversity, and changes in effector function (54, 55). Eventually, lymphoid cells are reconstituted by proliferation in the periphery (56, 57). The cellular composition of this renewed immune compartment has an impact in maintaining immune homeostasis and facing infectious insults.

Uncovering cellular and molecular signatures that correlate with improved outcomes may aid in the development of therapies designed to reduce the refractory period and improve overall outcomes post-sepsis. For example, transcriptional analysis has also been used to find associations with hospital-acquired illness in patients with chronic critical illness (58). Herein, we have employed scRNA-seq analysis to probe the lymphoid compartment, with a focus on differential cell responses associated with poor outcomes after sepsis. We compared healthy subjects, acutely (day 4 ± 1) septic patients, and septic patients at days 14-21 who either recovered rapidly or developed CCI. This comparison revealed unique differential expression patterns among CD8^+^ T cells, CD4^+^ T cells, NK cells, and B cells, which were subsequently used to conduct pathway enrichment analyses. We then focused on describing cell-type intrinsic differences between patients who rapidly recover and those that develop CCI. This approach revealed altered frequencies and unique cellular responses to sepsis across lymphoid cell types, and a transcriptomic signature of cell exhaustion in CD8^+^ TEM and a trend in NK cells in CCI. Overall, these results highlight cellular and molecular signatures of chronic critical illness and rapid recovery, while also emphasizing the importance of cell-specific functional analyses in understanding immune dynamics during sepsis.

The role of B cells in sepsis recovery is still emerging – an expansion in plasmablasts has been noted, with a CD39^high^ subset proposed to have a negative immunosuppressive role. However, B cell deficient mice exhibit poor survival, and provision of exogenous B cells to RAG deficient mice improves survival (10). These conflicting reports demonstrate the need for further assessment of this cell population in the outcome trajectory of septic subjects. Herein, we recapitulated the finding of plasmablast expansion in septic subjects, and note that these cells are a substantial proportion of total B cells in all septic donors. We also found that B cells displayed signs of a generally suppressed immune response. CCI vs rapid recovery comparison revealed downregulated processes in patients with CCI, including decreased cellular activation, differentiation and proliferation, decreased response to cell signaling, and decreased immune response in general (59, 60). Pathway analysis also suggested impaired cellular response to stress, decreased activation and proliferation, and abnormal MAPK signaling in B cells of patients with CCI, consistent with previous studies (61–63).

An innate immunity cell of lymphoid origin, NK cells have slowly emerged to be important to the host response to severe infections (64). Fewer NK cells have been previously reported in acutely septic patients compared to healthy subjects, similar to other lymphocytes (45). The observed decrease in day 4 ± 1 subjects aligns with these findings. Differential expression analysis revealed 168 DE genes in CCI subjects versus rapid recovery, with 156 up- and 12 downregulated. The differential gene expression profile highlighted continued activation in this cellular subset, with upregulated general activation as well as effector pathways including cytotoxicity, degranulation, and defense response. However, functional impairment was displayed in downregulation of pathways in bacterial, viral, and wounding response, and response to cytokines. Although we observed increased NK frequencies in CCI, we have previously demonstrated via scRNA-seq analysis that cytotoxic genes (*KLRC3*, *KLRC1*, and *KLRG1*) of NK cells were downregulated in patients with late sepsis (CCI and rapid recovery) (n=4), suggesting dysregulation of these cells (11). De Pablo et al. reported that septic patients with increased NK cell numbers have conversely lower survival probability (65) [similar results are seen in critically ill non-septic patients (66)]. Our findings indicate that, although the proportion of NK cells between these patients 2-3 weeks after sepsis were similar, they exhibited signs of impaired function in CCI. Dysregulation in the NK cell response to infection can either lead to hyper-inflammation shortly after sepsis onset or immunosuppression later on (67–69). These processes have been previously reported to be upregulated in NK cells during immunosuppression due to infection (53, 70). The NK cells in patients with CCI also exhibited a transcriptomic profile consistent with immune exhaustion.

Much of our analysis of CD4^+^ T cells, whose loss and dysfunction after sepsis are well understood to be related to patient mortality (54), revealed results similar to the published literature (12). Importantly, the comparison of CD4^+^ T cells in CCI versus rapid recovery sepsis survivors revealed 9 differentially upregulated genes in CCI that are associated with cell activation and proliferation (*CD4*, *CD27*, *CEBPB*, *DHPS*, *SLAMF1*, *LAPTM5*, *FADD*, *RASAL3*, and *TMIGD2*) (71). We noted altered cell frequencies in two CD4^+^ T subtypes, naive and central memory cells. Lymphopenia may explain the lower proportion of CD4^+^ T naive cells 4 days after sepsis onset (55, 72). Sepsis also causes global changes in expression of transcription factors that affect CD4^+^ T cell effector responses that we observed via pathway analysis. This includes a reduction in the ability to produce cytokines (45, 73, 74), reduced ability to proliferate (75), and induction of apoptosis (76). This impairment could affect the activation of these cells and the overall adaptive immune response against infections. The compromised ability of CD4^+^ T cells to coordinate immune responses and communicate with other immune cells could contribute to inadequate pathogen clearance and immune dysregulation, thereby impacting the severity and outcome of sepsis survivors. Although we did not see a significantly reduced proliferative capacity in these T cells consistent with T-cell exhaustion, there was notably decreased production of both TH_1_ and TH_2_ cytokines in CCI subjects. Importantly, lack of T cell IFN-γ production was distinctly reduced in the acute phase in those who would later be designated CCI, suggesting this may be a candidate assay for prognostic outcomes.

Although the importance of the CD4^+^ T cell response after sepsis has been recognized for some time (77), other host T cells responses have not traditionally been highlighted after severe infection, specifically in regard to sepsis survivors with poor outcomes. Pathway analysis of CD8^+^ T cells in chronic critical illness (CCI) versus rapid recovery patients revealed CD8^+^ T cells as activated and capable of sustaining effector functions and immune response. This highlights the complex interaction between innate and adaptive immunity after sepsis, as our previous publication showed that human septic MDSCs can suppress CD8^+^ T cell proliferation, but not CD4^+^ T cell proliferation in co-culture *ex vivo* (although both lymphocyte types had altered cytokine expression) (12). It should be noted, though, our current analyses also indicate some simultaneous reductions in inflammatory response pro-survival pathways in CD8^+^ T cells. Overall, the observed changes in these pathways indicates that CD8^+^ T cells in CCI patients are both metabolically active and functionally engaged in immune responses. However, indicators of chronic activation were present in scRNA-seq and functional outcomes *in vitro*, leading to assessment of exhaustion in this population. T cell exhaustion does not manifest uniformly, particularly in the understudied subacute response to surgical sepsis (8). Although increased in frequency as the dominant population among total CD8^+^ T cells, CD8^+^ TEM cells showed some signs of exhaustion in gene signature and cellular behavior, consistent with previous studies (78). We assessed for, and found, transcriptomic patterns of cellular exhaustion in CCI subjects in CD8^+^ TEM cells (downregulation of *TCF7* [transcription factor 7] and upregulation of *KLRG1* [killer cell lectin like receptor G1], *TIGIT*, *EOMES* [eomesodermin/Tbr2], *TOX*, *LAG3*, and *PDCD1* [programmed cell death 1]) (46).

Limitations of this study include its surgical sepsis patients coming from a single center. Although our treatment of sepsis aligns with Sepsis-3 guidelines, our exact protocols may differ from other centers, potentially impacting results. As scRNA-seq is performed on low cell numbers, the cell quantities are reported as proportional to the grouped dataset or parent lymphocyte population and are not directly quantitative. The observed changes in cell subset abundance were not reflective of longitudinal collection within subjects, preventing direct association with disease trajectory. Additionally, sampling limitations prevented the performance of scRNA-seq in conjunction with functional assays for direct comparison. Nevertheless, our cohort sample sizes are adequate when considering prior published literature and the amount of information generated via scRNA-seq, and, more importantly, contribute additional data to the broader community supporting larger integrated studies across centers.

## 5 Conclusion

Our study integrated different septic cohorts and cell annotation approaches at multiple levels of identification to reveal the dynamics of various lymphoid cell populations. Overall, the data generated support the concept of partial NK and T-cell exhaustion in patients with CCI, with loss of cytokine production and maintained proliferation. Distinct molecular signatures exist in sepsis patients with different clinical trajectories, emphasizing the importance of cell-specific time series analyses when attempting to both understand sepsis dynamics and design potential therapeutic interventions.

## Data Availability

The datasets presented in this study can be found in online repositories. The names of the repository/repositories and accession number(s) can be found below: GSE252331 (GEO).

## References

[1] Efron PA Brakenridge SC Mohr AM Barrios EL Polcz VE Anton S. The persistent inflammation, immunosuppression, and catabolism syndrome 10 years later. J Trauma Acute Care Surg. (2023) 95:790–9. doi: 10.1097/TA.0000000000004087 PMC10615691 37561664

[2] Darden DB Brakenridge SC Efron PA Ghita GL Fenner BP Kelly LS. Biomarker evidence of the persistent inflammation, immunosuppression and catabolism syndrome (PICS) in chronic critical illness (CCI) after surgical sepsis. Ann Surg. (2021) 274:664–73. doi: 10.1097/SLA.0000000000005067 PMC8592255 34506322

[3] Brakenridge SC Efron PA Cox MC Stortz JA Hawkins RB Ghita G. Current epidemiology of surgical sepsis: discordance between inpatient mortality and 1-year outcomes. Ann Surg. (2019) 270:502–10. doi: 10.1097/SLA.0000000000003458 PMC6942687 31356275

[4] Vanzant EL Lopez CM Ozrazgat-Baslanti T Ungaro R Davis R Cuenca AG. Persistent inflammation, immunosuppression, and catabolism syndrome after severe blunt trauma. J Trauma Acute Care Surg. (2014) 76:21–9; discussion 29-30. doi: 10.1097/TA.0b013e3182ab1ab5 24368353 PMC4310749

[5] Darden DB Ghita GL Wang Z Stortz JA Lopez MC Cox MC. Chronic critical illness elicits a unique circulating leukocyte transcriptome in sepsis survivors. J Clin Med. (2021) 10:3211. doi: 10.3390/jcm10153211 34361995 PMC8348105

[6] Goodwin AJ Ford DW. Readmissions among sepsis survivors: risk factors and prevention. Clin Pulm Med. (2018) 25:79–83. doi: 10.1097/CPM.0000000000000254 30237689 PMC6141202

[7] Chang DW Tseng CH Shapiro MF. Rehospitalizations following sepsis: common and costly. Crit Care Med. (2015) 43:2085–93. doi: 10.1097/CCM.0000000000001159 PMC5044864 26131597

[8] Yi JS Cox MA Zajac AJ. T-cell exhaustion: characteristics, causes and conversion. Immunology. (2010) 129:474–81. doi: 10.1111/j.1365-2567.2010.03255.x PMC2842494 20201977

[9] Singer M Deutschman CS Seymour CW Shankar-Hari M Annane D Bauer M. The third international consensus definitions for sepsis and septic shock (Sepsis-3). JAMA. (2016) 315:801–10. doi: 10.1001/jama.2016.0287 PMC4968574 26903338

[10] Dong X Tu H Qin S Bai X Yang F Li Z. Insights into the roles of B cells in patients with sepsis. J Immunol Res. (2023) 2023:7408967. doi: 10.1155/2023/7408967 37128298 PMC10148744

[11] Darden DB Dong X Brusko MA Kelly L Fenner B Rincon JC. A novel single cell RNA-seq analysis of non-myeloid circulating cells in late sepsis. Front Immunol. (2021) 12:696536. doi: 10.3389/fimmu.2021.696536 34484194 PMC8415415

[12] Barrios EL Leary JR Darden DB Rincon JC Willis M Polcz VE. The post-septic peripheral myeloid compartment reveals unexpected diversity in myeloid-derived suppressor cells. Front Immunol. (2024) 15:1355405. doi: 10.3389/fimmu.2024.1355405 38720891 PMC11076668

[13] Darden DB Bacher R Brusko MA Knight P Hawkins RB Cox MC. Single-cell RNA-seq of human myeloid-derived suppressor cells in late sepsis reveals multiple subsets with unique transcriptional responses: A pilot study. Shock. (2021) 55:587–95. doi: 10.1097/SHK.0000000000001671 PMC8019679 33021571

[14] Mathias B Delmas AL Ozrazgat-Baslanti T Vanzant EL Szpila BE Mohr AM. Human myeloid-derived suppressor cells are associated with chronic immune suppression after severe sepsis/septic shock. Ann Surg. (2017) 265:827–34. doi: 10.1097/SLA.0000000000001783 PMC5102824 27163951

[15] Hollen MK Stortz JA Darden D Dirain ML Nacionales DC Hawkins RB. Myeloid-derived suppressor cell function and epigenetic expression evolves over time after surgical sepsis. Crit Care. (2019) 23:355. doi: 10.1186/s13054-019-2628-x 31722736 PMC6854728

[16] Loftus TJ Mira JC Ozrazgat-Baslanti T Ghita GL Wang Z Stortz JA. Sepsis and Critical Illness Research Center investigators: protocols and standard operating procedures for a prospective cohort study of sepsis in critically ill surgical patients. BMJ Open. (2017) 7:e015136. doi: 10.1136/bmjopen-2016-015136 PMC5642775 28765125

[17] Zheng GX Terry JM Belgrader P Ryvkin P Bent ZW Wilson R. Massively parallel digital transcriptional profiling of single cells. Nat Commun. (2017) 8:14049. doi: 10.1038/ncomms14049 28091601 PMC5241818

[18] Andrews S. FastQC: a quality control tool for high throughput sequence data(2010). Available online at: http://www.bioinformatics.babraham.ac.uk/projects/fastqc (Accessed June 2024)

[19] Gaidatzis D Burger L Florescu M Stadler MB. Analysis of intronic and exonic reads in RNA-seq data characterizes transcriptional and post-transcriptional regulation. Nat Biotechnol. (2015) 33:722–9. doi: 10.1038/nbt.3269 26098447

[20] Srivastava A Malik L Sarkar H Patro R. A Bayesian framework for inter-cellular information sharing improves dscRNA-seq quantification. Bioinformatics. (2020) 36:i292–9. doi: 10.1093/bioinformatics/btaa450 PMC7355277 32657394

[21] Srivastava A Malik L Smith T Sudbery I Patro R. Alevin efficiently estimates accurate gene abundances from dscRNA-seq data. Genome Biol. (2019) 20:65. doi: 10.1186/s13059-019-1670-y 30917859 PMC6437997

[22] He D Zakeri M Sarkar H Soneson C Srivastava A Patro R. Alevin-fry unlocks rapid, accurate and memory-frugal quantification of single-cell RNA-seq data. Nat Methods. (2022) 19:316–22. doi: 10.1038/s41592-022-01408-3 PMC8933848 35277707

[23] R: A language and environment for statistical computing: R Core Team. (2022). Available online at: https://www.R-project.org/ (Accessed June 2024).

[24] Python Language Reference Delaware: Python Software Foundation (2019). Available online at: https://docs.python.org/3/ (Accessed May 2024).

[25] Zhu A Srivastava A Ibrahim JG Patro R Love MI. Nonparametric expression analysis using inferential replicate counts. Nucleic Acids Res. (2019) 47:e105. doi: 10.1093/nar/gkz622 31372651 PMC6765120

[26] Griffiths JA Richard AC Bach K Lun ATL Marioni JC. Detection and removal of barcode swapping in single-cell RNA-seq data. Nat Commun. (2018) 9:2667. doi: 10.1038/s41467-018-05083-x 29991676 PMC6039488

[27] Lun ATL Riesenfeld S Andrews T Dao TP Gomes T participants in the 1st Human Cell Atlas J. EmptyDrops: distinguishing cells from empty droplets in droplet-based single-cell RNA sequencing data. Genome Biol. (2019) 20:63. doi: 10.1186/s13059-019-1662-y 30902100 PMC6431044

[28] Murtagh F Legendre P. Ward’s hierarchical agglomerative clustering method: which algorithms implement Ward’s criterion? J Classif. (2014) 31:274–95. doi: 10.1007/s00357-014-9161-z

[29] Korsunsky I Millard N Fan J Slowikowski K Zhang F Wei K. Fast, sensitive and accurate integration of single-cell data with Harmony. Nat Methods. (2019) 16:1289–96. doi: 10.1038/s41592-019-0619-0 PMC6884693 31740819

[30] Leland McInnes JH Saul N Grossberger L. UMAP: uniform manifold approximation and projection. J Open Source Softw. (2018) 3:861. doi: 10.21105/joss.00861

[31] Blondel VD Guillaume J-L Lambiotte R Lefebvre E. Fast unfolding of communities in large networks. J Stat Mechanics: Theory Experiment. (2008) 2008:P10008. doi: 10.1088/1742-5468/2008/10/P10008

[32] Aran D Looney AP Liu L Wu E Fong V Hsu A. Reference-based analysis of lung single-cell sequencing reveals a transitional profibrotic macrophage. Nat Immunol. (2019) 20:163–72. doi: 10.1038/s41590-018-0276-y PMC6340744 30643263

[33] Martens JH Stunnenberg HG. BLUEPRINT: mapping human blood cell epigenomes. Haematologica. (2013) 98:1487–9. doi: 10.3324/haematol.2013.094243 PMC3789449 24091925

[34] Consortium EP. An integrated encyclopedia of DNA elements in the human genome. Nature. (2012) 489:57–74. doi: 10.1038/nature11247 22955616 PMC3439153

[35] Schmiedel BJ Singh D Madrigal A Valdovino-Gonzalez AG White BM Zapardiel-Gonzalo J. Impact of genetic polymorphisms on human immune cell gene expression. Cell. (2018) 175:1701–1715 e16. doi: 10.1016/j.cell.2018.10.022 30449622 PMC6289654

[36] Mabbott NA Baillie JK Brown H Freeman TC Hume DA. An expression atlas of human primary cells: inference of gene function from coexpression networks. BMC Genomics. (2013) 14:632. doi: 10.1186/1471-2164-14-632 24053356 PMC3849585

[37] Monaco G Lee B Xu W Mustafah S Hwang YY Carre C. RNA-seq signatures normalized by mRNA abundance allow absolute deconvolution of human immune cell types. Cell Rep. (2019) 26:1627–1640 e7. doi: 10.1016/j.celrep.2019.01.041 30726743 PMC6367568

[38] Novershtern N Subramanian A Lawton LN Mak RH Haining WN McConkey ME. Densely interconnected transcriptional circuits control cell states in human hematopoiesis. Cell. (2011) 144:296–309. doi: 10.1016/j.cell.2011.01.004 21241896 PMC3049864

[39] Hao Y Hao S Andersen-Nissen E Mauck WM 3rd Zheng S Butler A. Integrated analysis of multimodal single-cell data. Cell. (2021) 184:3573–3587 e29. doi: 10.1016/j.cell.2021.04.048 34062119 PMC8238499

[40] Benzanson J Edelman A Karpinski S Shah V. Julia: A fresh approach to numerical computing. SIAM Rev. (2017) 59:65–98. doi: 10.1137/141000671

[41] Zhou Y Zhou B Pache L Chang M Khodabakhshi AH Tanaseichuk O. Metascape provides a biologist-oriented resource for the analysis of systems-level datasets. Nat Commun. (2019) 10:1523. doi: 10.1038/s41467-019-09234-6 30944313 PMC6447622

[42] Hao Y Stuart T Kowalski MH Choudhary S Hoffman P Hartman A. Dictionary learning for integrative, multimodal and scalable single-cell analysis. Nat Biotechnol. (2024) 42:293–304. doi: 10.1038/s41587-023-01767-y 37231261 PMC10928517

[43] Finfer S Venkatesh B Hotchkiss RS Sasson SC. Lymphopenia in sepsis-an acquired immunodeficiency? Immunol Cell Biol. (2023) 101:535–44. doi: 10.1111/imcb.12611 36468797

[44] Adigbli D Liu R Meyer J Cohen J Di Tanna GL Gianacas C. Early persistent lymphopenia and risk of death in critically ill patients with and without sepsis. Shock. (2024) 61:197–203. doi: 10.1097/SHK.0000000000002284 38151771

[45] Boomer JS To K Chang KC Takasu O Osborne DF Walton AH. Immunosuppression in patients who die of sepsis and multiple organ failure. JAMA. (2011) 306:2594–605. doi: 10.1001/jama.2011.1829 PMC3361243 22187279

[46] Roe K. NK-cell exhaustion, B-cell exhaustion and T-cell exhaustion-the differences and similarities. Immunology. (2022) 166:155–68. doi: 10.1111/imm.v166.2 35266556

[47] Sanchez-Correa B Valhondo I Hassouneh F Lopez-Sejas N Pera A Bergua JM. DNAM-1 and the TIGIT/PVRIG/TACTILE axis: novel immune checkpoints for natural killer cell-based cancer immunotherapy. Cancers (Basel). (2019) 11:877. doi: 10.3390/cancers11060877 31234588 PMC6628015

[48] Miggelbrink AM Jackson JD Lorrey SJ Srinivasan ES Waibl-Polania J Wilkinson DS. CD4 T-cell exhaustion: does it exist and what are its roles in cancer? Clin Cancer Res. (2021) 27:5742–52. doi: 10.1158/1078-0432.CCR-21-0206 PMC8563372 34127507

[49] Tietscher S Wagner J Anzeneder T Langwieder C Rees M Sobottka B. A comprehensive single-cell map of T cell exhaustion-associated immune environments in human breast cancer. Nat Commun. (2023) 14:98. doi: 10.1038/s41467-022-35238-w 36609566 PMC9822999

[50] Roederer M. Interpretation of cellular proliferation data: avoid the panglossian. Cytometry A. (2011) 79:95–101. doi: 10.1002/cyto.a.v79a.2 21265003

[51] Ma C Liu H Yang S Li H Liao X Kang Y. The emerging roles and therapeutic potential of B cells in sepsis. Front Pharmacol. (2022) 13:1034667. doi: 10.3389/fphar.2022.1034667 36425582 PMC9679374

[52] Heidarian M Griffith TS Badovinac VP. Sepsis-induced changes in differentiation, maintenance, and function of memory CD8 T cell subsets. Front Immunol. (2023) 14:1130009. doi: 10.3389/fimmu.2023.1130009 36756117 PMC9899844

[53] Guo Y Patil NK Luan L Bohannon JK Sherwood ER. The biology of natural killer cells during sepsis. Immunology. (2018) 153:190–202. doi: 10.1111/imm.2018.153.issue-2 29064085 PMC5765373

[54] Martin MD Badovinac VP Griffith TS. CD4 T cell responses and the sepsis-induced immunoparalysis state. Front Immunol. (2020) 11:1364. doi: 10.3389/fimmu.2020.01364 32733454 PMC7358556

[55] Cabrera-Perez J Condotta SA Badovinac VP Griffith TS. Impact of sepsis on CD4 T cell immunity. J Leukoc Biol. (2014) 96:767–77. doi: 10.1189/jlb.5MR0114-067R PMC4197564 24791959

[56] Shankar-Hari M Fear D Lavender P Mare T Beale R Swanson C. Activation-associated accelerated apoptosis of memory B cells in critically ill patients with sepsis. Crit Care Med. (2017) 45:875–82. doi: 10.1097/CCM.0000000000002380 28296810

[57] Jensen IJ Li X McGonagill PW Shan Q Fosdick MG Tremblay MM. Sepsis leads to lasting changes in phenotype and function of memory CD8 T cells. Elife. (2021) 10:e70989. doi: 10.7554/eLife.70989 34652273 PMC8589447

[58] Chung KP Su JY Wang YF Budiarto BR Yeh YC Cheng JC. Immunometabolic features of natural killer cells are associated with infection outcomes in critical illness. Front Immunol. (2024) 15:1334882. doi: 10.3389/fimmu.2024.1334882 38426112 PMC10902670

[59] de Pablo R Monserrat J Prieto A Alvarez-Mon M. Role of circulating lymphocytes in patients with sepsis. BioMed Res Int. (2014) 2014:671087. doi: 10.1155/2014/671087 25302303 PMC4163419

[60] Davenport EE Burnham KL Radhakrishnan J Humburg P Hutton P Mills TC. Genomic landscape of the individual host response and outcomes in sepsis: a prospective cohort study. Lancet Respir Med. (2016) 4:259–71. doi: 10.1016/S2213-2600(16)00046-1 PMC4820667 26917434

[61] Wilson JK Zhao Y Singer M Spencer J Shankar-Hari M. Lymphocyte subset expression and serum concentrations of PD-1/PD-L1 in sepsis - pilot study. Crit Care. (2018) 22:95. doi: 10.1186/s13054-018-2020-2 29661225 PMC5902875

[62] Schenz J Tamulyte S Nusshag C Brenner T Poschet G Weigand MA. Population-specific metabolic alterations in professional antigen-presenting cells contribute to sepsis-associated immunosuppression. Shock. (2020) 53:5–15. doi: 10.1097/SHK.0000000000001337 31738315

[63] Monserrat J de Pablo R Diaz-Martin D Rodriguez-Zapata M de la Hera A Prieto A. Early alterations of B cells in patients with septic shock. Crit Care. (2013) 17:R105. doi: 10.1186/cc12750 23721745 PMC4056890

[64] Wang F Cui Y He D Gong L Liang H. Natural killer cells in sepsis: Friends or foes? Front Immunol. (2023) 14:1101918. doi: 10.3389/fimmu.2023.1101918 36776839 PMC9909201

[65] de Pablo R Monserrat J Torrijos C Martin M Prieto A Alvarez-Mon M. The predictive role of early activation of natural killer cells in septic shock. Crit Care. (2012) 16:413. doi: 10.1186/cc11204 22405329 PMC3681341

[66] Andaluz-Ojeda D Iglesias V Bobillo F Nocito M Loma AM Nieto C. Early levels in blood of immunoglobulin M and natural killer cells predict outcome in nonseptic critically ill patients. J Crit Care. (2013) 28:1110 e7–1110 e10. doi: 10.1016/j.jcrc.2013.06.007 23953491

[67] Mithal LB Arshad M Swigart LR Khanolkar A Ahmed A Coates BM. Mechanisms and modulation of sepsis-induced immune dysfunction in children. Pediatr Res. (2022) 91:447–53. doi: 10.1038/s41390-021-01879-8 PMC9752201 34952937

[68] Otto GP Sossdorf M Claus RA Rodel J Menge K Reinhart K. The late phase of sepsis is characterized by an increased microbiological burden and death rate. Crit Care. (2011) 15:R183. doi: 10.1186/cc10332 21798063 PMC3387626

[69] Hamers L Kox M Pickkers P. Sepsis-induced immunoparalysis: mechanisms, markers, and treatment options. Minerva Anestesiol. (2015) 81:426–39. 24878876

[70] Liu D Huang SY Sun JH Zhang HC Cai QL Gao C. Sepsis-induced immunosuppression: mechanisms, diagnosis and current treatment options. Mil Med Res. (2022) 9:56. doi: 10.1186/s40779-022-00422-y 36209190 PMC9547753

[71] Provost E Weier CA Leach SD. Multiple ribosomal proteins are expressed at high levels in developing zebrafish endoderm and are required for normal exocrine pancreas development. Zebrafish. (2013) 10:161–9. doi: 10.1089/zeb.2013.0884 PMC3673614 23697888

[72] Hoser GA Skirecki T Zlotorowicz M Zielinska-Borkowska U Kawiak J. Absolute counts of peripheral blood leukocyte subpopulations in intraabdominal sepsis and pneumonia-derived sepsis: a pilot study. Folia Histochem Cytobiol. (2012) 50:420–6. doi: 10.5603/FHC.2012.0057 23042273

[73] Roth G Moser B Krenn C Brunner M Haisjackl M Almer G. Susceptibility to programmed cell death in T-lymphocytes from septic patients: a mechanism for lymphopenia and Th2 predominance. Biochem Biophys Res Commun. (2003) 308:840–6. doi: 10.1016/S0006-291X(03)01482-7 12927795

[74] Heidecke CD Hensler T Weighardt H Zantl N Wagner H Siewert JR. Selective defects of T lymphocyte function in patients with lethal intraabdominal infection. Am J Surg. (1999) 178:288–92. doi: 10.1016/S0002-9610(99)00183-X 10587185

[75] Cabrera-Perez J Condotta SA James BR Kashem SW Brincks EL Rai D. Alterations in antigen-specific naive CD4 T cell precursors after sepsis impairs their responsiveness to pathogen challenge. J Immunol. (2015) 194:1609–20. doi: 10.4049/jimmunol.1401711 PMC4412277 25595784

[76] Ammer-Herrmenau C Kulkarni U Andreas N Ungelenk M Ravens S Hubner C. Sepsis induces long-lasting impairments in CD4+ T-cell responses despite rapid numerical recovery of T-lymphocyte populations. PloS One. (2019) 14:e0211716. doi: 10.1371/journal.pone.0211716 30730978 PMC6366777

[77] Hotchkiss RS Opal S. Immunotherapy for sepsis–a new approach against an ancient foe. N Engl J Med. (2010) 363:87–9. doi: 10.1056/NEJMcibr1004371 PMC4136660 20592301

[78] Condotta SA Rai D James BR Griffith TS Badovinac VP. Sustained and incomplete recovery of naive CD8+ T cell precursors after sepsis contributes to impaired CD8+ T cell responses to infection. J Immunol. (2013) 190:1991–2000. doi: 10.4049/jimmunol.1202379 23355736 PMC3578009

