# Supplementary figures and images for: Unique lymphocyte transcriptomic profiles in septic patients with chronic critical illness
Source: Front Immunol. 2024 Dec 3;15:1478471. doi: 10.3389/fimmu.2024.1478471 (PMC11649506; doi:10.3389/fimmu.2024.1478471)

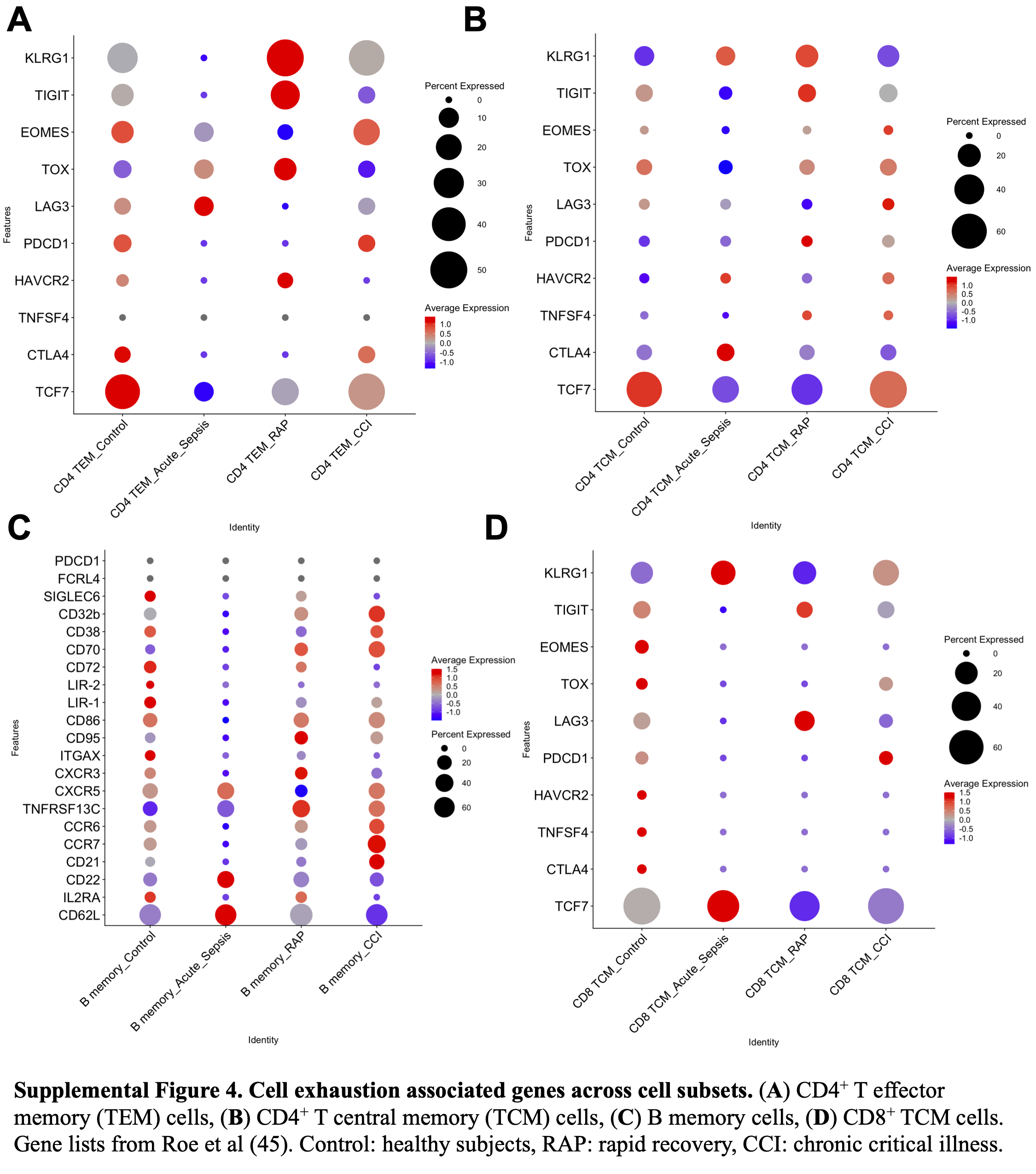

Supplement: Supplementary file 1 [file Image1.tiff]

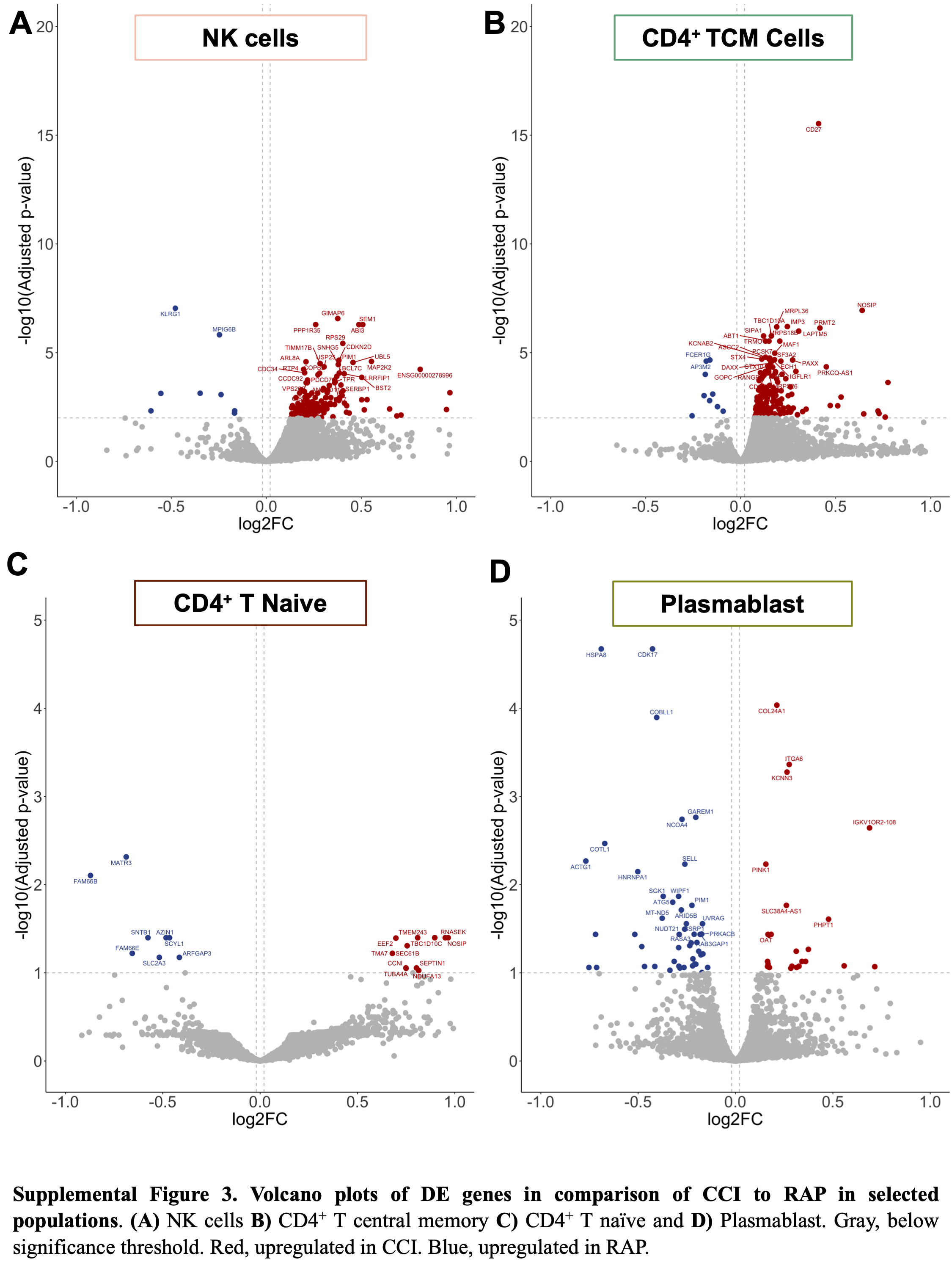

Supplement: Supplementary file 2 [file Image2.tiff]

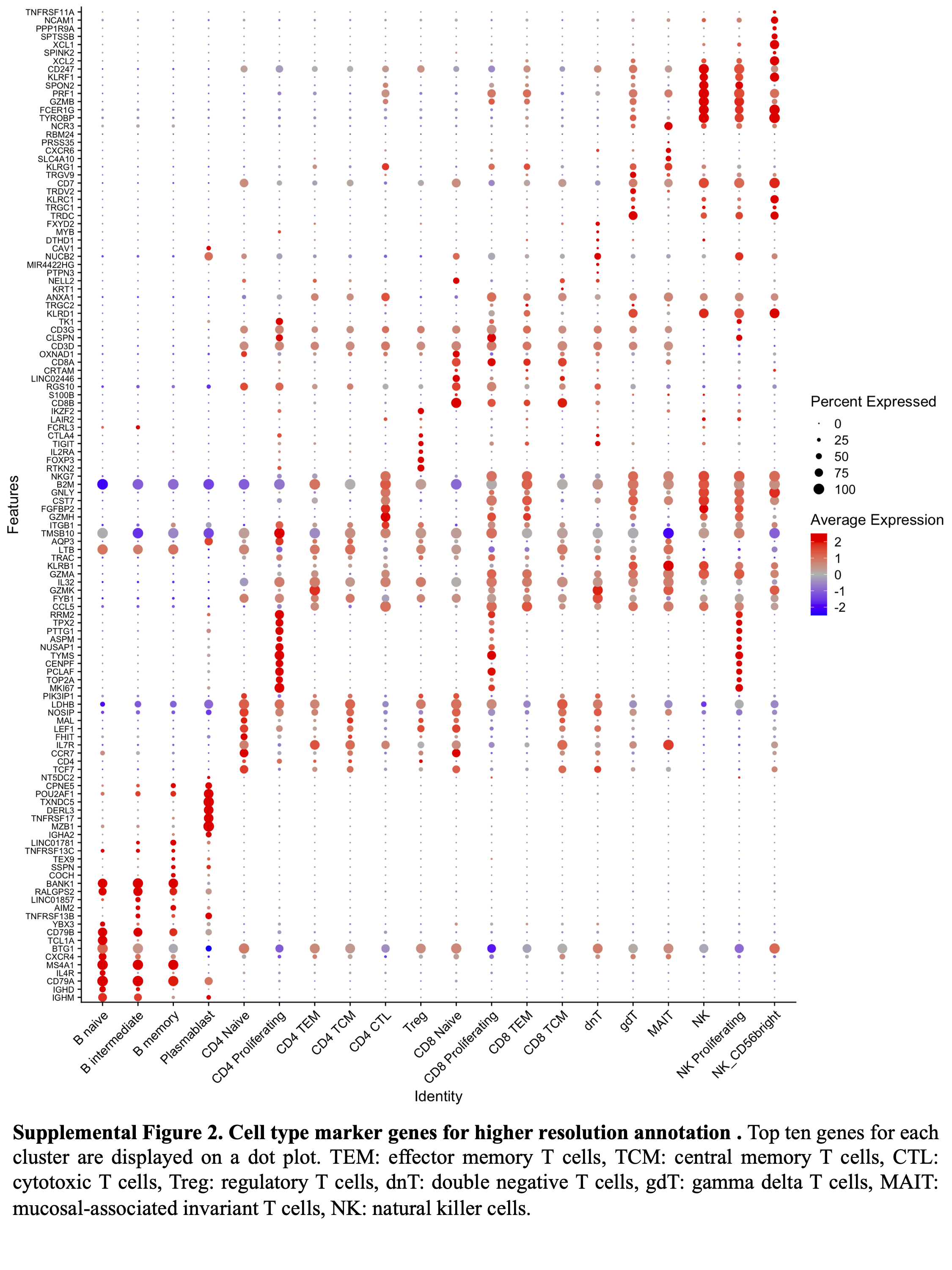

Supplement: Supplementary file 3 [file Image3.tiff]

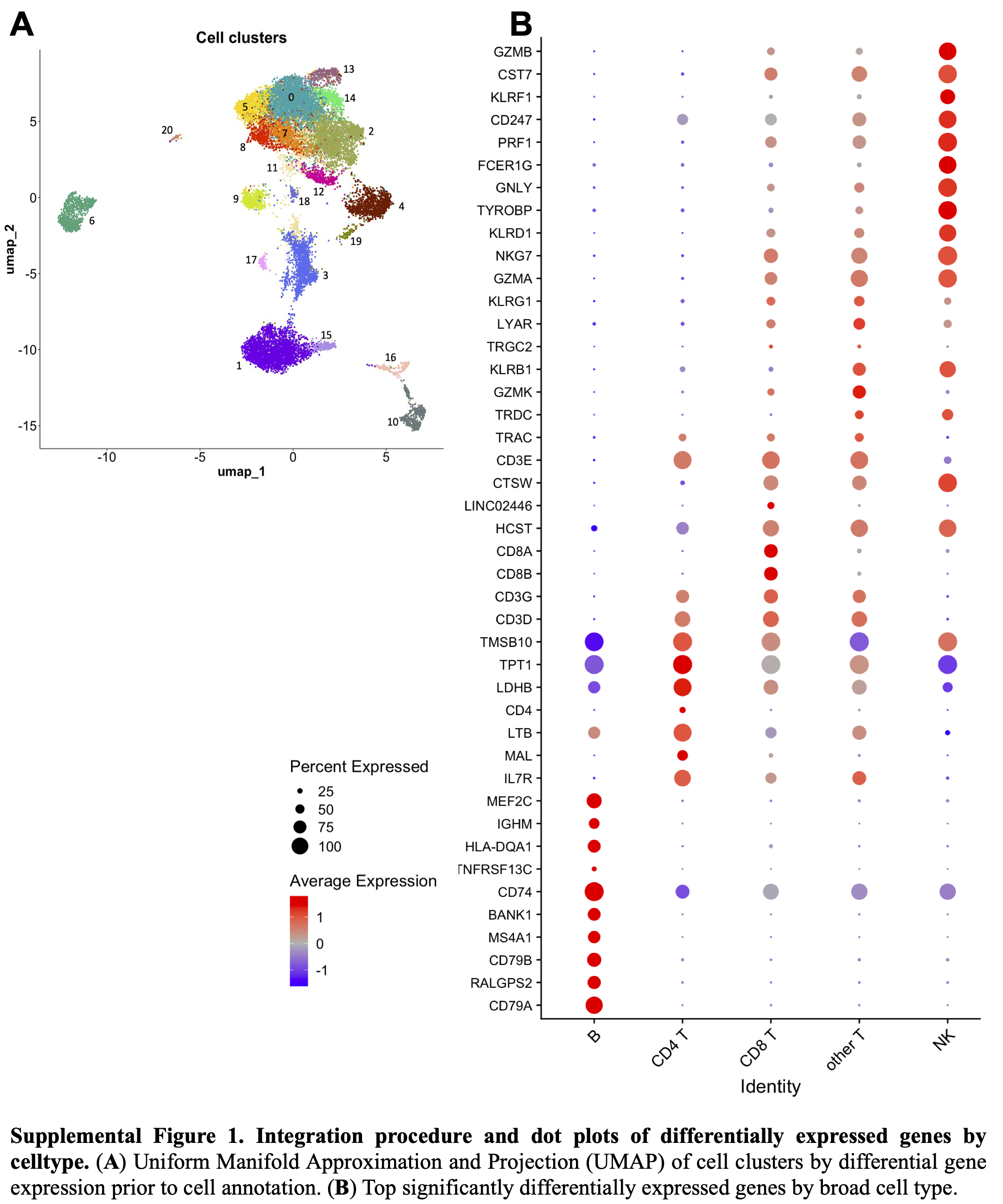

Supplement: Supplementary file 4 [file Image4.tiff]
